# Joint associations of lung function of both general and abdominal obesity with cardiometabolic multimorbidity: a cross-sectional study

**DOI:** 10.3389/fmed.2026.1761219

**Published:** 2026-03-10

**Authors:** Shuyan Wan, Mengmeng Shi, Yue Gao

**Affiliations:** 1 Department of Health Education, Jinhua Municipal Central Hospital, Zhejiang, China; 2 Hemodialysis Center, The Second People’s Hospital of Jiashan, Jiaxing, China

**Keywords:** abdominal obesity, cardiometabolic multimorbidity, lung function, obesity, restrictive spirometry pattern

## Abstract

**Background:**

Cardiometabolic multimorbidity (CMM) has become an escalating public health challenge amid global population aging. Although lung function has been implicated in individual cardiometabolic conditions, its role in predicting CMM, particularly in relation to obesity, remains poorly characterized. This study aimed to evaluate the associations between lung function indicators and CMM, and to explore their joint associations with general and abdominal obesity.

**Methods:**

This cross-sectional study included 35,414 community-dwelling adults aged ≥65 years from the Zhejiang Provincial Chronic Obstructive Lung Disease Screening in Jinhua City, China. Lung function was assessed by standardized spirometry. Participants were classified as having preserved lung function or restrictive spirometry (FEV₁/FVC ≥ 0.70 with FVC%predicted <80%). General obesity was defined as BMI ≥ 28.0 kg/m^2^, and abdominal obesity as waist circumference ≥90 cm (men) or ≥85 cm (women). CMM was defined as coexistence of ≥2 conditions: type 2 diabetes, coronary heart disease, or stroke. Multivariable logistic regression models examined associations of lung function and obesity with CMM, adjusting for demographic, lifestyle, and clinical factors. Joint effects and dose–response relationships were assessed.

**Results:**

CMM prevalence was 1.02%. In fully adjusted models, higher lung function was inversely associated with CMM. Compared to the lowest quartile, the highest quartile had significantly lower CMM odds for FVC%predicted (OR = 0.60, 95%CI: 0.44, 0.81), FEV₁%predicted (OR = 0.71, 95%CI: 0.53, 0.95), and PEF%predicted (OR = 0.64, 95%CI: 0.47, 0.86). Restrictive spirometry was associated with 56% higher CMM odds versus preserved lung function. Both general obesity (OR = 1.30 per SD, 95%CI: 1.18, 1.43) and abdominal obesity (OR = 1.33 per SD, 95%CI: 1.21, 1.45) significantly increased CMM odds. Joint analysis showed participants with restrictive spirometry and abdominal obesity had the highest CMM, while those with preserved lung function and normal abdominal circumference had 65% lower odds (OR = 0.35, 95%CI: 0.25, 0.49).

**Conclusion:**

These findings suggest that reduced lung function in combination with obesity represents a high-risk phenotype that is more strongly associated with CMM. Simultaneous screening of lung function and obesity status may help identify older adults at greatest risk for CMM. However, longitudinal studies are warranted to confirm the temporality of these associations.

## Introduction

With the global population aging at an accelerating pace, the coexistence of multiple chronic diseases, particularly cardiometabolic multimorbidity (CMM), has emerged as a major public health challenge (1, 2). CMM refers to the simultaneous presence of two or more cardiometabolic conditions in an individual, typically including type 2 diabetes, coronary heart disease (CHD), and stroke (3). It is estimated that the negative impact of CMM on life expectancy far exceeds that of any single disease, potentially shortening lifespan by more than 50% (4). Moreover, individuals with CMM have a 3.7 to 6.9 times higher risk of mortality compared to those without these conditions (5). Data from a nationally representative survey suggest that CMM among Chinese older adults has shown a marked upward trend, increasing from 11.6 to 16.9%, with further growth expected (6). CMM is also associated with a higher likelihood of severe disability, decreased quality of life, and increased complications, all of which contribute substantially to personal and societal socioeconomic burdens (7, 8). In light of this growing epidemic, identifying and understanding the determinants of CMM is crucial for developing effective prevention strategies.

Lung function is a key indicator of respiratory health (9). While traditional risk factors such as poor diet, physical inactivity, and obesity have been widely established in relation to cardiometabolic diseases (10), the role of lung function as a potential predictor of CMM remains under-investigated. Existing evidence suggests that impaired lung function is linked to systemic inflammation, metabolic abnormalities, and elevated cardiovascular mortality. However, most previous studies have focused on individual disease outcomes rather than CMM as a composite endpoint. Understanding the relationship between lung function and CMM is particularly important given that older adults are commonly burdened by multiple coexisting chronic conditions rather than isolated diseases. Furthermore, lung function and cardiometabolic disorders share common risk factors, including obesity, physical inactivity, and systemic inflammation, suggesting potential interconnected pathways. Both obstructive and restrictive lung impairments may contribute to the development of CMM through chronic low-grade inflammation and metabolic disturbances (11–13). In addition, obesity, particularly abdominal obesity, not only increases mechanical load on the lung but may also aggravate metabolic disturbances through multiple mechanisms, including adipose tissue dysfunction with excessive release of pro-inflammatory adipokines (e.g., TNF-*α*, IL-6), insulin resistance, altered adiponectin secretion, and lipotoxicity, creating a vicious cycle (14–16). To date, the interaction between lung function and CMM in the context of obesity remains poorly understood.

To address these knowledge gaps, our study based on data from the Zhejiang Provincial Chronic Obstructive lung Disease Screening and Risk Assessment, aimed to evaluate the associations between three key lung function indicators, including forced vital capacity (FVC), forced expiratory volume in one second (FEV₁), and peak expiratory flow (PEF), and the prevalence of CMM. Furthermore, we examined the impact of different lung function patterns (normal, restrictive) and investigated the joint effects of lung function and both general and abdominal obesity on the risk of CMM. Stratified analyses were also conducted to assess potential dose–response relationships between lung function and CMM across obesity categories. The findings of this study may offer novel insights into the joint associations of lung function and obesity (both general and abdominal) with CMM risk, thereby supporting the early identification of high-risk populations and the development of integrated prevention strategies that simultaneously address respiratory function, metabolic health, and obesity management.

## Methods

### Study population

This study was a population-based cross-sectional study based on the data from the Zhejiang Provincial Chronic Obstructive lung Disease Screening and Risk Assessment, in Jinhua City from May to December 2022. Participants were recruited through a multi-stage cluster sampling method from community-dwelling residents within the target jurisdiction. The initial sample included 60,886 participants aged 65 years and above from Jinhua municipality. For the current analysis, participants were excluded based on the following criteria: (1) incomplete lung function data (n = 9,530); (2) missing obesity-related indicators (including BMI and waist circumference) (n = 3,635); and (3) incomplete cardiometabolic multimorbidity information (including data on type 2 diabetes, CHD, and stroke) (*n* = 12,307). After applying these exclusion criteria, the final analytical sample comprised 35,414 participants. The study protocol was approved by the Ethics Committee of Jinhua Central Hospital (Approval No: (Research) 2023-Ethical Review-67). Informed consent was obtained from all participants through standardized procedures administered by local healthcare institutions.

### Assessment of lung function

Lung function was assessed using standardized portable spirometry devices (model: PF280, manufacturer: U-Breath, Hangzhou, Zhejiang, China) operated by trained health professionals. The same device model was used consistently across all screening sites in Jinhua City. Participants performed at least three acceptable forced expiratory maneuvers, from which the best effort was retained for analysis. Participants performed at least three acceptable forced expiratory maneuvers in accordance with the Chinese National Guidelines of Lung Function Test (17). Acceptable maneuvers were defined as: (1) satisfactory start with no hesitation and good explosive effort; (2) smooth expiratory curve without interruption, cough, air leak, tongue or tooth obstruction of the mouthpiece, or glottis closure; (3) expiratory time ≥6 s or a plateau of at least 1 s in the volume-time curve; and (4) at least three but no more than eight repetitions, with the difference between the best and second-best values of FVC and FEV₁ ≤ 0.15 L. The best effort was retained for analysis.

The spirometric parameters analyzed included FVC, FEV₁, and PEF. These lung function indices were further divided into quartiles (Q1-Q4) according to their distribution in the total sample for dose–response analyses. Additionally, according to the Chinese expert consensus on standardization of adult lung function diagnosis (18), participants were classified into two categories: preserved Lung function (normal FEV₁ and FVC with no airflow limitation) and restrictive spirometry (FEV₁/FVC ≥ 0.70 with FVC% pred < 80%). To enable international comparisons, we additionally calculated lung function Z-scores using the Global Lung Function Initiative (GLI-2012) reference equations for Asian populations (19) and examined their associations with CMM.

### Assessment of obesity and abdominal obesity

General obesity was assessed using body mass index (BMI), calculated as weight in kilograms divided by height in meters squared (kg/m^2^). In accordance with cut-off points recommended for the Chinese population by the Working Group on Obesity in China (WGOC), participants were categorized as having normal weight (BMI < 24.0 kg/m^2^), overweight (BMI 24.0–27.9 kg/m^2^), or obesity (BMI ≥ 28.0 kg/m^2^) (20). Abdominal obesity was determined using waist circumference (WC), with thresholds set at ≥90 cm for men and ≥85 cm for women, based on criteria established by the Chinese Diabetes Society to reflect elevated cardiometabolic risk (21).

### Cardiometabolic multimorbidity

CMM (3) was defined as the coexistence of two or more of the following physician-diagnosed conditions: (1) Type 2 Diabetes Mellitus-defined as self-reported physician diagnosis; (2) Stroke defined as self-reported history of cerebrovascular accident confirmed by a physician. (3) CHD-including self-reported diagnosis of myocardial infarction, angina, or other ischemic heart conditions confirmed by a physician. This definition aligns with the established consensus framework for CMM, which specifically identifies diabetes, heart disease, and stroke as the core cardiometabolic conditions (3). Those with none or only one of the conditions were classified as not having CMM. To assess the robustness of our findings, we conducted a sensitivity analysis using an expanded definition of CMM that included hypertension as a fourth component condition (2).

### Potential covariates

Several potential confounding factors were selected based on their established or plausible effects on lung function, obesity, and CMM. Demographic variables included age (continuous), sex (male/female), education level (primary school or below vs. junior high school or above), occupation (categorized as agricultural work, housework, retired, or others), and per capita household income (<¥50,000 or ≥¥50,000 annually). Lifestyle factors comprised smoking status (yes/no) and occupational exposure to hazardous gases (yes/no). Clinical measurements included systolic and diastolic blood pressure (SBP and DBP, mmHg), resting heart rate (beats per minute), and peripheral oxygen saturation (SpO₂, %). All covariates were obtained through standardized physical examinations and structured interviews conducted by trained fieldworkers using validated protocols.

### Statistical analysis

Participants were grouped by CMM status for descriptive statistics. Baseline characteristics were described using mean ± standard deviation (SD) for continuous variables and frequency (percentage) for categorical variables. Differences between groups were compared utilizing one-way Analysis of Variance (ANOVA) or Chi-square tests. To determine whether lung function (FVC%pred, FEV1%pred, and PEF%pred) and CMM are associated, logistic regression models were used. Before model fitting, we confirmed that there was no multicollinearity among all the covariates (the variance inflation factor [VIF] of all covariates was less than 5). Covariates were selected based on established literature identifying key risk factors for both impaired lung function and cardiometabolic conditions (22–25), including demographic factors (age, sex), socioeconomic indicators (education, occupation, income per capita), lifestyle factors (smoking status), occupational exposures (hazardous gases), anthropometric measures (obesity, abdominal obesity), and cardiovascular parameters (SBP, DBP, heart rate, and SpO_2_). We constructed three models with progressively more comprehensive covariate adjustment. The Model 1 was run without covariate adjustment, Model 2 was constructed with adjustments for age and sex, and Model 3 adjusted for Model 2 plus education, occupation, income per capita, smoking status, occupational exposure to hazardous gases, obesity, abdominal obesity, SBP, DBP, Heart rate, and SpO_2_. Odds ratios (ORs) and 95% confidence intervals (CIs) were calculated for each quartile of lung function parameters, using the lowest quartile (Q1) as the reference. To assess the robustness of our findings to the choice of cut-points, we conducted sensitivity analyses using tertiles and quintiles of lung function parameters. In addition, *P* for trend was estimated for checking a linear trend in multivariable models. For lung function categories (preserved, restrictive), restrictive function served as the reference group. To facilitate comparisons across different metrics and with previous studies, we also modeled lung function parameters (FVC%pred, FEV₁%pred, and PEF%pred) and obesity indicators (BMI and WC) as continuous variables per standard deviation (SD) increase. These analyses provide estimates of the change in CMM risk associated with a one-SD increment in each exposure, complementing the categorical analyses using quartiles, tertiles, and quintiles.

To explore the joint effects of lung function and obesity on CMM, we created combined exposure groups by cross-classifying participants according to their lung function category (preserved lung function vs. restrictive spirometry) and obesity status (normal weight vs. overweight/obesity for general obesity; normal vs. abdominal obesity for abdominal obesity). For each combined analysis, participants with the theoretically highest risk phenotype, restrictive spirometry with overweight/obesity (for general obesity) and restrictive spirometry with abdominal obesity (for abdominal obesity), were set as the reference group. Logistic regression models were used to estimate the odds ratios for CMM across the combined categories, with adjustment for the same covariates as in the primary analyses. Multiplicative interactions between lung function category and obesity were assessed by including product terms in the models, and the *P* for interaction values are reported. Restricted cubic spline (RCS) regression models with three knots were used to evaluate the dose–response relationships between lung function indicators (FVC%pred, FEV1%pred, and PEF%pred) and the risk of CMM, stratified by general and abdominal obesity status. For the primary analysis, knots were placed at the 10th, 50th, and 90th percentiles. A sensitivity analysis was used alternative knot placements at the 25th, 50th, and 75th percentiles yielded. All models were adjusted for the same covariates as in the primary logistic regression analyses.

All analyses were performed in R 4.5.1 (http://www.r-project.org) with two-sided *p* < 0.05 considered statistically significant.

## Results

### Characteristics of the participants

A total of 35,413 participants were included in the final analysis. Table 1 presents the baseline characteristics of participants stratified by lung function category (preserved lung function vs. restrictive spirometry), general obesity (normal vs. overweight/obesity), and abdominal obesity (normal vs. abdominal obesity). By lung function category, 22.4% of participants were classified as having restrictive spirometry. Compared to those with preserved lung function, participants with restrictive spirometry were significantly older (67.80 vs. 67.30 years), more likely to be male (62.5% vs. 54.1%), less educated (70.2% vs. 63.5% with primary education or less), and had higher smoking rates (44.5% vs. 38.4%). They also exhibited lower FVC%pred (66.9% vs. 101.8%), FEV₁%pred (71.2% vs. 103.9%), and PEF%pred (62.6% vs. 85.9%) (all *p* < 0.001). By obesity status, 40.4% of participants were classified as overweight/obese, and 26.3% had abdominal obesity. As expected, individuals with overweight/obesity or abdominal obesity had significantly higher BMI, WC, SBP, and DBP compared to their normal counterparts (all *p* < 0.001). Notably, FVC%pred was slightly lower in the overweight/obese group compared to normal weight participants (92.6% vs. 94.9%, *p* < 0.001). Characteristics stratified by quartiles of FVC%pred, FEV₁%pred, and PEF%pred are presented in Supplementary Tables S1–S3.

**Table 1:** Descriptive characteristics of the participants.

Characteristics	Lung function category		*P*	Obesity		*P*	Abdominal obesity		*P*
	Preserved lung function	Restrictive spirometry		Normal	Overweight/Obesity		Normal	Abdominal obesity	
N (%)	27,484 (77.6)	7,930 (22.4)		21,121 (59.6)	14,293 (40.4)		26,106 (73.7)	9,308 (26.3)	
Age, years	67.30 (4.8)	67.80 (4.6)	<0.001	67.68 (4.58)	67.02 (4.89)	<0.001	67.38 (4.77)	67.51 (4.58)	0.026
Sex			<0.001			<0.001			<0.001
Male	14,862 (54.1)	4,960 (62.5)		11,367 (53.8)	8,455 (59.2)		15,062 (57.7)	4,760 (51.1)	
Female	12,622 (45.9)	2,970 (37.5)		9,754 (46.2)	5,838 (40.8)		11,044 (42.3)	4,548 (48.9)	
Education			<0.001			<0.001			0.794
Primary or less	17,443 (63.5)	5,567 (70.2)		14,048 (66.5)	8,962 (62.7)		16,973 (65.0)	6,037 (64.9)	
Junior high or above	10,041 (36.5)	2,363 (29.8)		7,073 (33.5)	5,331 (37.3)		9,133 (35.0)	3,271 (35.1)	
Occupation			<0.001			<0.001			<0.001
Agriculture	16,376 (59.6)	4,409 (55.6)		12,649 (59.9)	8,136 (56.9)		15,369 (58.9)	5,416 (58.2)	
Housework	2,654 (9.7)	611 (7.7)		1946 (9.2)	1,319 (9.2)		2,259 (8.7)	1,006 (10.8)	
Retired	5,017 (18.3)	1,676 (21.1)		3,844 (18.2)	2,849 (19.9)		5,123 (19.6)	1,570 (16.9)	
Others	3,437 (12.5)	1,234 (15.6)		2,682 (12.7)	1989 (13.9)		3,355 (12.9)	1,316 (14.1)	
Income per capita (¥)			0.004			<0.001			<0.001
<50, 000	17,324 (63.0)	5,140 (64.8)		13,768 (65.2)	8,696 (60.8)		16,741 (64.1)	5,723 (61.5)	
≥50, 000	10,160 (37.0)	2,790 (35.2)		7,353 (34.8)	5,597 (39.2)		9,365 (35.9)	3,585 (38.5)	
Smoking status			<0.001			<0.001			0.236
Yes	10,561 (38.4)	3,526 (44.5)		8,121 (38.4)	5,966 (41.7)		10,433 (40.0)	3,654 (39.3)	
No	16,923 (61.6)	4,404 (55.5)		13,000 (61.6)	8,327 (58.3)		15,673 (60.0)	5,654 (60.7)	
Occupational exposure to hazardous gases			<0.001			0.039			0.010
Yes	3,909 (14.2)	864 (10.9)		2,781 (13.2)	1992 (13.9)		3,445 (13.2)	1,328 (14.3)	
No	23,575 (85.8)	7,066 (89.1)		18,340 (86.8)	12,301 (86.1)		22,661 (86.8)	7,980 (85.7)	<0.001
BMI (kg/m^2^)	23.4 (3.0)	23.7 (3.3)	<0.001	21.5 (1.8)	26.4 (2.2)		22.61 (2.6)	25.96 (2.9)	
WC (cm)	82.4 (8.5)	83.4 (9.1)	<0.001	79.1 (7.2)	87.68 (8.2)	<0.001	79.0 (6.2)	92.7 (6.4)	
SBP (mmHg)	131.4 (13.8)	132.0 (14.2)	<0.001	130.5 (14.1)	133.03 (13.5)	<0.001	130.6 (13.6)	134.03 (14.3)	<0.001
DBP (mmHg)	77.4 (9.4)	77.5 (9.9)	0.715	77.0 (9.6)	78.16 (9.4)	<0.001	76.9 (9.4)	79.0 (9.5)	<0.001
Heart rate (times/min)	74.9 (9.8)	74.8 (10.2)	0.822	74.7 (10.2)	75.17 (9.6)	<0.001	74.6 (9.9)	75.63 (10.0)	<0.001
SpO_2_ (%)	96.01 (5.6)	95.4 (5.5)	<0.001	95.8 (5.7)	95.95 (5.4)	0.124	95.8 (5.7)	96.00 (5.2)	0.034
FVC%pred	101.80 (32.0)	66.9 (14.2)	<0.001	94.9 (37.1)	92.64 (23.8)	<0.001	94.6 (34.7)	92.28 (25.0)	<0.001
FEV_1_%pred	103.94 (58.5)	71.2 (17.0)	<0.001	97.5 (66.8)	95.18 (24.5)	<0.001	96.9 (23.9)	95.77 (97.1)	0.077
PEF%pred	85.94 (25.9)	62.6 (126.9)	<0.001	80.8 (81.5)	80.53 (25.3)	0.622	80.9 (74.1)	80.03 (25.6)	0.222

BMI, body mass index; WC, waist circumference; SBP, systolic blood pressure; DBP, diastolic blood pressure; SpO₂, peripheral oxygen saturation; FVC%pred, forced vital capacity percent predicted; FEV₁%pred, forced expiratory volume in 1 s percent predicted; PEF%pred, peak expiratory flow percent predicted.

Values were means ± SD or n (percentages).

CMM prevalence was 1.02% (361 participants). Detailed characteristics stratified by CMM status are provided in Supplementary Table S4. Compared to included participants, those excluded were significantly older, more likely to be male, less educated, engaged in agriculture, and current smokers, with lower BMI and WC but higher blood pressure (all *p* < 0.05), while no significant differences were observed for lung function parameters (Supplementary Table S5).

### Associations of lung function, lung function categories and CMM

We used multivariable logistic regression models to evaluate the associations between quartiles of lung function parameters (FVC%pred, FEV₁%pred, and PEF%pred) and the odds of CMM in Table 2. For FVC%pred, individuals in the highest quartile (Q4) had 40% lower odds of having CMM compared to those in the lowest quartile (Q1) (OR = 0.60; 95% CI: 0.44, 0.81, *P* for trend <0.001) in the fully adjusted model. Similarly, FEV₁%pred showed an inverse association with CMM, with those in Q4 having significantly lower odds compared to Q1 (OR = 0.71; 95% CI: 0.53, 0.95, *P* for trend = 0.006). PEF%pred also showed an inverse association with CMM, with those in Q4 having significantly lower odds compared to Q1 (OR = 0.64; 95% CI: 0.47, 0.86, *P* for trend = 0.005). Sensitivity analyses using tertiles and quintiles of FVC%pred, FEV₁%pred, and PEF%pred yielded results consistent with the primary quartile-based analyses, with higher lung function categories consistently associated with lower odds of CMM across all models (Supplementary Tables S6, S7). When modeled as continuous variables, each S increase in lung function parameters was significantly associated with lower odds of CMM. In the fully adjusted model, the odds ratios per SD increase were 0.75 (95% CI, 0.64, 0.88) for FVC%pred, 0.71 (95% CI, 0.55, 0.92) for FEV₁%pred, and 0.67 (95% CI, 0.50, 0.90) for PEF%pred. In sensitivity analyses using GLI-derived Z-scores, both FVC Z-score and FEV₁ Z-score were significantly associated with lower odds of CMM across all models in Supplementary Table S8.

**Table 2:** Associations of lung function and lung function category with cardiometabolic multimorbidity.

Lung function	Case/Total	Model 1	Model 2	Model 3	Model 4
		OR (95%CI)	OR (95%CI)	OR (95%CI)	OR (95%CI)
FVC%pred					
Quartile1	128/8865	Reference	Reference	Reference	Reference
Quartile2	94/8842	**0.73 (0.56, 0.96)**	0.77 (0.59, 1.01)	**0.75 (0.57, 0.98)**	0.77 (0.58, 1.00)
Quartile3	71/8857	**0.55 (0.41, 0.74)**	**0.58 (0.43, 0.78)**	**0.57 (0.43, 0.77)**	**0.60 (0.45, 0.80)**
Quartile4	68/8850	**0.53 (0.39, 0.71)**	**0.57 (0.42, 0.76)**	**0.56 (0.41, 0.75)**	**0.60 (0.44, 0.81)**
*P* for trend		**<0.001**	**<0.001**	**<0.001**	**<0.001**
Per-SD		**0.71 (0.61, 0.83)**	**0.73 (0.63, 0.86)**	**0.73 (0.62, 0.85)**	**0.75 (0.64, 0.88)**
FEV_1_%pred					
Quartile1	114/8855	Reference	Reference	Reference	Reference
Quartile2	70/8884	0.89 (0.68, 1.16)	0.92 (0.70, 1.20)	0.90 (0.69, 1.18)	0.94 (0.72, 1.23)
Quartile3	75/8824	**0.61 (0.45, 0.82)**	**0.64 (0.47, 0.86)**	**0.63 (0.46, 0.84)**	**0.67 (0.49, 0.90)**
Quartile4	119/8851	**0.66 (0.49, 0.88)**	**0.66 (0.49, 0.89)**	**0.66 (0.49, 0.89)**	**0.71 (0.53, 0.95)**
*P* for trend		**<0.001**	**<0.001**	**0.001**	**0.006**
Per, SD		**0.67 (0.52, 0.86)**	**0.68 (0.53, 0.87)**	**0.68 (0.53, 0.87)**	**0.71 (0.55, 0.92)**
PEF%pred					
Quartile1	119/8858	Reference	Reference	Reference	Reference
Quartile2	86/8854	**0.72 (0.54, 0.95)**	**0.75 (0.57, 0.99)**	**0.75 (0.56, 0.99)**	**0.75 (0.57, 0.99)**
Quartile3	87/8848	**0.73 (0.55, 0.96)**	0.79 (0.60, 1.04)	0.77 (0.58, 1.02)	0.78 (0.59, 1.03)
Quartile4	69/8854	**0.58 (0.43, 0.77)**	**0.66 (0.48, 0.88)**	**0.63 (0.46, 0.85)**	**0.64 (0.47, 0.86)**
*P* for trend		**<0.001**	**0.007**	**0.004**	**0.005**
Per-SD		**0.60 (0.46, 0.80)**	**0.69 (0.52, 0.91)**	**0.67 (0.50, 0.89)**	**0.67 (0.50, 0.90)**
Lung function category					
Restrictive spirometry	242/27484	Reference	Reference	Reference	Reference
Preserved lung function	119/7930	**0.58 (0.47, 0.73)**	**0.62 (0.50, 0.77)**	**0.61 (0.49, 0.76)**	**0.64 (0.51, 0.80)**

Model 1: adjust for none; Model 2: adjust for age and sex; Model 3: adjust for Model 2 plus education, occupation, income per capita, smoking status, occupational exposure to hazardous gases; Model 4: adjust for Model 3 plus obesity, abdominal obesity, SBP, DBP, Heart rate, and SpO_2_. *p*-values which are lower than the statistical significance of 0.05 have been bolded.

In Table 2, we further examined the association between lung function categories and CMM. Using restrictive lung function as the reference group, preserved lung function was significantly associated with increased odds of CMM (OR = 0.64; 95% CI: 0.51, 0.80). Across all models, restrictive spirometry consistently exhibited a strong independent association with higher CMM risk.

### Associations of general and abdominal obesity and CMM

Both general and abdominal obesity were significantly associated with increased odds of CMM in Table 3. In Model 4, compared to participants with obesity, those who were overweight (OR = 0.46; 95% CI: 0.33, 0.64) or normal weight (OR = 0.67; 95% CI: 0.48, 0.95) had significantly lower odds of CMM. When analyzed as a continuous variable, each SD increase in BMI was associated with 30% higher odds of CMM (OR = 1.30; 95% CI: 1.18, 1.43). For abdominal obesity, participants without abdominal obesity had significantly lower odds of CMM compared to those with abdominal obesity (OR = 0.55; 95% CI: 0.44, 0.69) in Model 4. Each SD increase in waist circumference was associated with 33% higher odds of CMM (OR = 1.33; 95% CI: 1.21, 1.45).

**Table 3:** Associations of general obesity and abdominal obesity with cardiometabolic multimorbidity.

Obesity	Case/Total	Model 1	Model 2	Model 3	Model 3
		OR (95%CI)	OR (95%CI)	OR (95%CI)	OR (95%CI)
General obesity					
Obesity	142/11680	Reference	Reference	Reference	Reference
Overweight	46/2613	**0.46 (0.34, 0.65)**	**0.44 (0.32, 0.62)**	**0.45 (0.32, 0.63)**	**0.46 (0.33, 0.64)**
Normal	173/21121	**0.69 (0.50, 0.97)**	**0.67 (0.48, 0.95)**	**0.66 (0.47, 0.93)**	**0.67 (0.48, 0.95)**
BMI (Per-SD)		**1.30 (1.18, 1.43)**	**1.32 (1.20, 1.45)**	**1.31 (1.19, 1.44)**	**1.30 (1.18, 1.43)**
Abdominal obesity					
Yes	141/9308	Reference	Reference	Reference	Reference
No	220/26106	**0.55 (0.45, 0.68)**	**0.54 (0.44, 0.67)**	**0.55 (0.45, 0.69)**	**0.55 (0.44, 0.69)**
WC (Per-SD)		**1.37 (1.25, 1.49)**	**1.34 (1.23, 1.47)**	**1.33 (1.21, 1.45)**	**1.33 (1.21, 1.45)**

Model 1: adjust for none; Model 2: adjust for age and sex; Model 3: adjust for Model 2 plus education, occupation, income per capita, smoking status, occupational exposure to hazardous gases; Model 4: adjust for Model 3 plus Lung function category, SBP, DBP, Heart rate, and SpO_2_.

General obesity: Normal (18.5 kg/m^2^ ≤ BMI < 24.0 kg/m^2^); overweight (24.0 kg/m^2^ ≤ BMI < 28.0 kg/m^2^); obesity (BMI ≥ 28.0 kg/m^2^).

### Joint effects of lung function category and obesity

We further examined the joint effects of impaired lung function and obesity on the risk of CMM in Table 4. For general obesity, using participants with restrictive spirometry and overweight/obesity as the reference group, those with preserved lung function and normal weight had the lowest odds of CMM (OR = 0.41; 95% CI: 0.30, 0.57), followed by those with preserved lung function and overweight/obesity (OR = 0.73; 95% CI: 0.54, 0.99) and those with restrictive spirometry and normal weight (OR = 0.78; 95% CI: 0.54, 1.12). For abdominal obesity, using participants with restrictive spirometry and abdominal obesity as the reference, those with preserved lung function and normal abdominal circumference had the lowest odds of CMM (OR = 0.35; 95% CI: 0.25, 0.49), followed by those with restrictive spirometry and normal abdominal circumference (OR = 0.56; 95% CI: 0.39, 0.82) and those with preserved lung function and abdominal obesity (OR = 0.64; 95% CI: 0.45, 0.92). The interaction between lung function category and obesity was not statistically significant for either general obesity (*P* for interaction = 0.157) or abdominal obesity (*P* for interaction = 0.916). In sensitivity analyses using an expanded CMM definition that included hypertension as a fourth component condition, the results remained consistent with the primary findings across all analyses, including the associations of lung function parameters with CMM (Supplementary Table S9), the associations of obesity indicators with CMM (Supplementary Table S10), and the joint effects of lung function and obesity on CMM (Supplementary Table S11).

**Table 4:** Joint effect of lung function category and obesity on cardiometabolic multimorbidity.

Lung function category	Obesity	Case/Total	OR (95%CI)	*P* for interaction
Lung function category + General obesity				0.157
Restrictive spirometry	Overweight and obesity	59/3421	Reference	
Restrictive spirometry	Normal	60/4509	0.78 (0.54, 1.12)	
Preserved Lung function	Overweight and obesity	129/10872	**0.73 (0.54, 0.99)**	
Preserved Lung function	Normal	133/16612	**0.41 (0.30, 0.57)**	
Lung function category + Abdominal obesity				0.916
Restrictive spirometry	Abdominal obesity	50/2295	Reference	
Restrictive spirometry	Normal	69/5635	**0.56 (0.39, 0.82)**	
Preserved Lung function	Abdominal obesity	91/7013	**0.64 (0.45, 0.92)**	
Preserved Lung function	Normal	151/20471	**0.35 (0.25, 0.49)**	

Adjust for sex, age, education, occupation, income per capita, smoking status, occupational exposure to hazardous gases, obesity, abdominal obesity, SBP, DBP, Heart rate, and SpO_2_. *p*-values which are lower than the statistical significance of 0.05 have been bolded.

### Restricted cubic spline analyses

**Figure 1:**
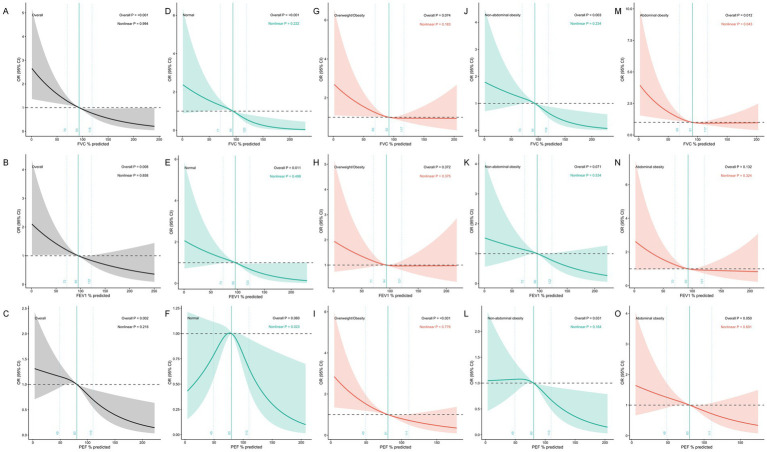
Restricted cubic spline regressions of OR and 95% CI of the association of lung function with cardiometabolic multimorbidity by obesity. **(A)** FVC%pred and CMM among overall participants. **(B)** FEV_1_%pred and CMM among overall participants. **(C)** PEF%pred and CMM among overall participants. **(D)** FVC%pred and CMM among participants with normal. **(E)** FEV_1_%pred and CMM among participants with normal. **(F)** PEF%pred and CMM among participants with normal. **(G)** FVC%pred and CMM among participants with overweight/obesity. **(H)** FEV_1_%pred and CMM among participants with overweight/obesity. **(I)** PEF%pred and CMM among participants with overweight/obesity. **(J)** FVC%pred and CMM among participants with non-abdominal obesity. **(K)** FEV_1_%pred and CMM among participants with non-abdominal obesity. **(L)** PEF%pred and CMM among participants with non-abdominal obesity. **(M)** FVC%pred and CMM among participants with abdominal obesity. **(N)** FEV_1_%pred and CMM among participants with abdominal obesity. **(O)** PEF%pred and CMM among participants with abdominal obesity. Solid lines represent estimated ORs; shaded areas represent 95% confidence intervals. All models were adjusted for age, sex, education, occupation, income per capita, smoking status, occupational exposure to hazardous gases, systolic and diastolic blood pressure, heart rate, and SpO_2_. *p* values for overall and nonlinearity are provided in each panel. The reference value was set at the median of the lowest quartile of lung function values.

To further assess the dose–response relationship between lung function and CMM, we applied RCS regression models, stratified by general and abdominal obesity status (Figure 1). In the overall analysis, all three lung function parameters showed significant inverse associations with CMM risk in Figures 1A–C. For FVC%pred, the overall association was significant (*P* overall < 0.001). For FEV₁%pred, the overall association was significant (*P* overall = 0.008). For PEF%pred, the overall association was significant (*P* overall = 0.002). Among normal weight participants in Figures 1D–F, significant inverse associations were observed for FVC%pred (*P* overall < 0.001) and FEV₁%pred (*P* overall = 0.011), while PEF%pred showed evidence of nonlinearity (*P* for nonlinearity = 0.023). Among overweight/obesity participants in Figures 1G–I, only PEF%pred demonstrated a significant association with CMM (*P* overall < 0.001). Among participants without abdominal obesity in Figures 1J–L, significant inverse associations were found for FVC%pred (*P* overall = 0.003) and PEF%pred (*P* overall = 0.031). Among those with abdominal obesity in Figures 1M–O, FVC%pred showed a significant association (*P* overall = 0.012) with evidence of nonlinearity (*P* for nonlinearity = 0.043). Sensitivity analyses using alternative knot placements (25th, 50th, and 75th percentiles) yielded consistent results in Supplementary Figure S1.

## Discussion

In this large-scale, nationally representative study involving over 42,000 Chinese individuals, our findings demonstrate a robust and independent association between impaired lung function and the risk of cardiometabolic multimorbidity (CMM). Specifically, lower predicted values of forced vital capacity (FVC%pred) and forced expiratory volume in one second (FEV1%pred) were significantly associated with higher odds of CMM. Notably, restrictive spirometry was consistently linked to increased CMM risk. Furthermore, we identified a significant interaction between impaired lung function and both general and abdominal obesity, suggesting a convergence of respiratory and metabolic dysfunction as a critical pathway in the development of multimorbidity. Importantly, this study not only focuses on metabolic factors in CMM prevention but also highlights lung function as a potential integrative biomarker reflecting overall systemic health.

Our results contribute several important insights into the relationship between lung function and CMM. Both FVC%pred and FEV1%pred exhibited strong inverse associations with CMM odds. These findings align with previous cohort studies showing that lower FVC and FEV1 are linked with elevated risks of cardiovascular and metabolic diseases (26, 27). While FVC and FEV1 are traditionally used to characterize restrictive (28), respectively, they also serve as broader indicators of lung parenchymal integrity and general lung health in the general population (29). The lung parenchyma, with its dense capillary network, is highly susceptible to cumulative insults from low-grade systemic inflammation, oxidative stress, and microvascular injury—all key pathological mechanisms driving CMM (30, 31). It is worth noting that the association between FVC%pred and CMM did not demonstrate a strictly linear trend, possibly indicating a threshold effect or influence from unmeasured confounders such as physical activity levels or early-life lung development. Conversely, PEF%pred did not show a statistically significant association with CMM, implying that peak flow, a measure of large airway caliber and effort-dependent performance, may be less sensitive than volume-based indices like FVC and FEV1 in predicting multisystem metabolic derangement (22, 32, 33). Collectively, these findings underscore the potential clinical utility of lung function—particularly FVC and FEV1—as screening tools for early identification of individuals at high risk for CMM.

This study also enriches our understanding of the associations between specific lung function categories and CMM risk. We observed a consistently strong relationship between restrictive spirometry and elevated CMM risk, using preserved lung function as the reference. This consistent association across models suggests that restrictive ventilatory impairment may hold unique pathophysiological relevance in the context of multimorbidity. Restrictive impairment, often characterized by reduced lung volume, has been closely linked to low-grade systemic inflammation, insulin resistance, and metabolic syndrome—all contributors to endothelial dysfunction and atherosclerosis, thereby increasing the likelihood of cardiometabolic comorbidities (31). Restrictive patterns may also reflect systemic conditions such as obesity, interstitial lung disease, or chronic kidney disease—each of which are independently associated with increased CMM risk (34).

Furthermore, our findings demonstrate that both general and abdominal obesity are independently associated with significantly higher odds of CMM. These results confirm the detrimental effects of both total and central adiposity in the pathogenesis of multimorbidity (4). The strong association between obesity and CMM may be driven by multiple intertwined pathophysiological pathways. On one hand, excess adipose tissue, especially visceral fat, secretes proinflammatory cytokines (e.g., TNF-*α*, IL-6) and adipokines, fostering chronic low-grade inflammation and insulin resistance, which together facilitate the development of multiple cardiometabolic disorders (35). On the other hand, abdominal obesity more directly reflects visceral fat accumulation and is closely linked to dyslipidemia, hypertension, and glucose intolerance. Several studies have shown that WC is a better predictor than BMI for metabolic syndrome and type 2 diabetes (36). In our study, both BMI and WC were independently associated with CMM, underscoring the value of incorporating both general and abdominal obesity assessments in CMM risk stratification. Clinically and from a public health perspective, these findings emphasize the importance of considering both body weight and body fat distribution in disease prevention strategies. Future studies should investigate the long-term impact of distinct obesity phenotypes (e.g., metabolically healthy vs. unhealthy obesity) on CMM risk and evaluate whether interventions targeting weight reduction and waist control can effectively prevent multimorbidity.

To our knowledge, this is the first study to explore the joint effects of lung function impairment and obesity on CMM risk. Our results indicate a significant synergistic interaction between impaired lung function and obesity. In joint analyses, individuals with both restrictive lung impairment and general obesity had the highest risk of CMM. Similarly, the combination of restrictive lung impairment and abdominal obesity also significantly increased CMM risk. These findings suggest a multiplicative rather than additive effect of impaired lung function and obesity on cardiometabolic burden. Restrictive lung dysfunction is often accompanied by reduced lung volumes and impaired diffusing capacity, potentially reflecting systemic inflammation, oxidative stress, and interstitial remodeling—processes that are also central to insulin resistance, endothelial dysfunction, and metabolic dysregulation (37). Obesity, particularly visceral fat accumulation, further aggravates these pathways through the secretion of inflammatory cytokines (e.g., TNF-*α*, IL-6) and free fatty acids, contributing to a pro-inflammatory and pro-atherogenic state (38). The coexistence of both impairments may amplify the inflammatory–metabolic axis, leading to accelerated CMM development. Previous studies have shown that abdominal obesity is independently associated with reduced lung function, and that such reductions may precede the onset of metabolic syndrome or diabetes (26, 39). Our findings highlight the importance of concurrent screening for lung function and adiposity in the assessment of CMM risk. Future research should focus on elucidating the molecular pathways underlying the lung–obesity interaction and investigating whether integrated interventions targeting both lung function and body composition can more effectively mitigate the progression of multimorbidity.

Despite the strengths of our study, including a large sample size and comprehensive covariate adjustment, several limitations should be acknowledged. First, the cross-sectional design precludes causal inference and introduces the potential for reverse causation. Although our findings demonstrate clear associations, the temporal sequence between impaired lung function and the onset of CMM cannot be established. Therefore, longitudinal studies are needed to clarify the directionality of these associations. Second, the definition of CMM and certain covariates (e.g., stroke and ischemic heart disease) relied on self-reported physician diagnoses, which may be subject to recall bias. Future studies could address this limitation by linking survey data to objective sources such as electronic health records, insurance claim databases, or disease registries, which would provide more accurate ascertainment of disease status and enable validation of self-reported diagnoses. Third, despite adjusting for a broad range of confounders, residual confounding due to unmeasured or imperfectly measured variables cannot be excluded, including physical activity (40–42) and diet (43, 44) and so on. Future research should incorporate these measures using standardized, validated instruments to better isolate the independent effects of lung function on CMM. Lastly, our study population was recruited from Jinhua City, an economically developed region in Eastern China, and consisted exclusively of Chinese middle-aged and older adults. Therefore, caution is warranted when generalizing our findings to less developed regions within China, other ethnic groups, younger populations, or settings with different healthcare systems and environmental exposures. The external validity of our results remains to be verified in future multi-center and cross-ethnic studies. Fifth, while our findings demonstrate significant associations between lung function, obesity, and CMM, the underlying biological mechanisms remain unexplored in this epidemiological study. We did not measure molecular biomarkers or investigate specific pathophysiological pathways that could mechanistically link these conditions. Future mechanistic research should examine specific molecular pathways and biomarkers to elucidate the biological basis of these associations. Promising avenues include: (1) systemic inflammation markers: high-sensitivity CRP, IL-6, TNF-*α* as potential mediators, given that both restrictive spirometry and obesity are associated with chronic low-grade inflammation (45, 46); (2) oxidative stress biomarkers (malondialdehyde, 8-isoprostane) and antioxidant capacity to assess whether oxidative damage pathways mediate the lung function-CMM relationship (47).

## Conclusion

In conclusion, our study demonstrates that reduced lung function, particularly restrictive lung function, is significantly associated with higher odds of CMM among Chinese older adults. These associations are especially pronounced among individuals with general or abdominal obesity, suggesting that the combination of impaired lung function and obesity may identify a particularly high-risk phenotype. Our findings highlight the potential value of incorporating routine lung function assessment into cardiometabolic risk stratification strategies for older adults in China. Future longitudinal research is needed to establish the temporal sequence and causal pathways linking lung function decline with CMM development, to investigate potentially reversible mechanisms, and to determine whether interventions targeting lung function or obesity can reduce CMM incidence. Such evidence would inform the development of integrated prevention strategies to address the growing burden of multimorbidity in aging populations.

## Data Availability

The raw data supporting the conclusions of this article will be made available by the corresponding author upon reasonable request.
