# Supplementary material for: Joint associations of lung function of both general and abdominal obesity with cardiometabolic multimorbidity: a cross-sectional study
Source: Front Med (Lausanne). 2026 Mar 10;13:1761219. doi: 10.3389/fmed.2026.1761219 (PMC13008965; doi:10.3389/fmed.2026.1761219)

Table S1. Characteristics of Participants by Quartiles of FVC%pred

|  | Q1 (Lowest) | Q2 | Q3 | Q4 (Highest) | *P* |
| --- | --- | --- | --- | --- | --- |
| N | 8865 | 8842 | 8857 | 8850 |  |
| Age, years | 67.76 (4.70) | 67.27 (4.70) | 67.19 (4.77) | 67.43 (4.69) | <0.001 |
| Sex |  |  |  |  | <0.001 |
| Male | 5536 (62.4) | 5095 (57.6) | 5016 (56.6) | 4175 (47.2) |  |
| Female | 3329 (37.6) | 3747 (42.4) | 3841 (43.4) | 4675 (52.8) |  |
| Education |  |  |  |  | <0.001 |
| Primary or less | 6203 (70.0) | 5590 (63.2) | 5501 (62.1) | 5716 (64.6) |  |
| Junior high or above | 2662 (30.0) | 3252 (36.8) | 3356 (37.9) | 3134 (35.4) |  |
| Occupation |  |  |  |  | <0.001 |
| Agriculture | 4941 (55.7) | 5093 (57.6) | 5275 (59.6) | 5476 (61.9) |  |
| Housework | 677 (7.6) | 771 (8.7) | 804 (9.1) | 1013 (11.4) |  |
| Retired | 1863 (21.0) | 1799 (20.3) | 1730 (19.5) | 1301 (14.7) |  |
| Others | 1384 (15.6) | 1179 (13.3) | 1048 (11.8) | 1060 (12.0) |  |
| Occupational exposure to hazardous gases |  |  |  |  | <0.001 |
| Yes | 977 (11.0) | 1224 (13.8) | 1226 (13.8) | 1346 (15.2) |  |
| No | 7888 (89.0) | 7618 (86.2) | 7631 (86.2) | 7504 (84.8) |  |
| Income per capita (¥) |  |  |  |  | 0.017 |
| <50, 000 | 5728 (64.6) | 5632 (63.7) | 5578 (63.0) | 5526 (62.4) |  |
| ≥50, 000 | 3137 (35.4) | 3210 (36.3) | 3279 (37.0) | 3324 (37.6) |  |
| Smoking status |  |  |  |  | <0.001 |
| Yes | 3911 (44.1) | 3646 (41.2) | 3611 (40.8) | 2919 (33.0) |  |
| No | 4954 (55.9) | 5196 (58.8) | 5246 (59.2) | 5931 (67.0) |  |
| SBP (mmHg) | 132.09 (14.23) | 131.81 (13.83) | 131.31 (14.07) | 130.99 (13.65) | <0.001 |
| DBP (mmHg) | 77.61 (9.90) | 77.65 (9.39) | 77.54 (9.64) | 77.15 (9.22) | 0.001 |
| Heart rate (times/min) | 74.88 (10.20) | 74.90 (9.89) | 74.86 (9.82) | 74.92 (9.95) | 0.98 |
| SpO_2_(%) | 95.51 (5.47) | 95.92 (5.56) | 96.09 (5.40) | 96.06 (6.06) | <0.001 |
| BMI (kg/m^2^) | 23.69 (3.29) | 23.63 (3.11) | 23.45 (2.99) | 23.18 (3.04) | <0.001 |
| WC (cm) | 83.42 (9.15) | 83.17 (8.84) | 82.49 (8.51) | 81.25 (8.24) | <0.001 |
| Obesity |  |  |  |  | <0.001 |
| Normal | 5041 (56.9) | 5102 (57.7) | 5335 (60.2) | 5643 (63.8) |  |
| Overweight | 802 (9.0) | 733 (8.3) | 566 (6.4) | 512 (5.8) |  |
| Obesity | 3022 (34.1) | 3007 (34.0) | 2956 (33.4) | 2695 (30.5) |  |
| Abdominal obesity |  |  |  |  | <0.001 |
| Normal | 6313 (71.2) | 6308 (71.3) | 6638 (74.9) | 6847 (77.4) |  |
| Abdominal Obesity | 2552 (28.8) | 2534 (28.7) | 2219 (25.1) | 2003 (22.6) |  |

Abbreviation: BMI, body mass index; WC, waist circumference; SBP, systolic blood pressure; DBP, diastolic blood pressure; SpO₂, peripheral oxygen saturation; FVC%pred, forced vital capacity percent predicted; FEV₁%pred, forced expiratory volume in 1 second percent predicted; PEF%pred, peak expiratory flow percent predicted.

Values were means ± SD or n (percentages).

Table S2. Characteristics of Participants by Quartiles of FEV_1_%pred

|  | Q1 (Lowest) | Q2 | Q3 | Q4 (Highest) | *P* |
| --- | --- | --- | --- | --- | --- |
| N | 8855 | 8884 | 8824 | 8851 | <0.001 |
| Age, years | 67.50 (4.77) | 67.15 (4.85) | 67.10 (4.75) | 67.90 (4.46) |  |
| Sex |  |  |  |  | <0.001 |
| Male | 5358 (60.5) | 5192 (58.4) | 5049 (57.2) | 4223 (47.7) |  |
| Female | 3497 (39.5) | 3692 (41.6) | 3775 (42.8) | 4628 (52.3) |  |
| Education |  |  |  |  | <0.001 |
| Primary or less | 6043 (68.2) | 5543 (62.4) | 5539 (62.8) | 5885 (66.5) |  |
| Junior high or above | 2812 (31.8) | 3341 (37.6) | 3285 (37.2) | 2966 (33.5) |  |
| Occupation |  |  |  |  | <0.001 |
| Agriculture | 5114 (57.8) | 5185 (58.4) | 5127 (58.1) | 5359 (60.5) |  |
| Housework | 668 (7.5) | 792 (8.9) | 830 (9.4) | 975 (11.0) |  |
| Retired | 1716 (19.4) | 1718 (19.3) | 1706 (19.3) | 1553 (17.5) |  |
| Others | 1357 (15.3) | 1189 (13.4) | 1161 (13.2) | 964 (10.9) |  |
| Occupational exposure to hazardous gases |  |  |  |  | <0.001 |
| Yes | 1066 (12.0) | 1235 (13.9) | 1212 (13.7) | 1260 (14.2) |  |
| No | 7789 (88.0) | 7649 (86.1) | 7612 (86.3) | 7591 (85.8) |  |
| Income per capita (¥) |  |  |  |  | 0.569 |
| <50, 000 | 5641 (63.7) | 5630 (63.4) | 5547 (62.9) | 5646 (63.8) |  |
| ≥50, 000 | 3214 (36.3) | 3254 (36.6) | 3277 (37.1) | 3205 (36.2) |  |
| Smoking status |  |  |  |  | <0.001 |
| Yes | 3907 (44.1) | 3734 (42.0) | 3610 (40.9) | 2836 (32.0) |  |
| No | 4948 (55.9) | 5150 (58.0) | 5214 (59.1) | 6015 (68.0) |  |
| SBP (mmHg) | 132.11 (14.09) | 131.85 (13.97) | 131.19 (13.94) | 131.04 (13.78) | <0.001 |
| DBP (mmHg) | 77.50 (9.80) | 77.62 (9.44) | 77.57 (9.56) | 77.27 (9.37) | 0.068 |
| Heart rate (times/min) | 74.97 (10.25) | 74.87 (9.82) | 74.77 (9.65) | 74.95 (10.13) | 0.555 |
| SpO_2_(%) | 95.53 (5.72) | 95.95 (5.68) | 95.98 (5.57) | 96.12 (5.55) | <0.001 |
| BMI (kg/m^2^) | 23.73 (3.29) | 23.56 (3.07) | 23.40 (2.96) | 23.26 (3.12) | <0.001 |
| WC (cm) | 83.57 (9.17) | 82.95 (8.68) | 82.30 (8.56) | 81.51 (8.36) | <0.001 |
| Obesity |  |  |  |  | <0.001 |
| Normal | 4961 (56.0) | 5274 (59.4) | 5381 (61.0) | 5505 (62.2) |  |
| Overweight | 815 (9.2) | 696 (7.8) | 532 (6.0) | 570 (6.4) |  |
| Obesity | 3079 (34.8) | 2914 (32.8) | 2911 (33.0) | 2776 (31.4) |  |
| Abdominal obesity |  |  |  |  | <0.001 |
| Normal | 6194 (69.9) | 6503 (73.2) | 6658 (75.5) | 6751 (76.3) |  |
| Abdominal Obesity | 2661 (30.1) | 2381 (26.8) | 2166 (24.5) | 2100 (23.7) |  |

Abbreviation: BMI, body mass index; WC, waist circumference; SBP, systolic blood pressure; DBP, diastolic blood pressure; SpO₂, peripheral oxygen saturation; FVC%pred, forced vital capacity percent predicted; FEV₁%pred, forced expiratory volume in 1 second percent predicted; PEF%pred, peak expiratory flow percent predicted.

Values were means ± SD or n (percentages).

Table S3. Characteristics of Participants by Quartiles of PEF%pred

|  | Q1 (Lowest) | Q2 | Q3 | Q4 (Highest) | *P* |
| --- | --- | --- | --- | --- | --- |
| N | 8858 | 8854 | 8848 | 8854 |  |
| Age, years | 68.06 (4.42) | 67.45 (4.71) | 67.10 (4.80) | 67.04 (4.87) | <0.001 |
| Sex |  |  |  |  | <0.001 |
| Male | 5588 (63.1) | 5474 (61.8) | 5034 (56.9) | 3726 (42.1) |  |
| Female | 3270 (36.9) | 3380 (38.2) | 3814 (43.1) | 5128 (57.9) |  |
| Education |  |  |  |  | <0.001 |
| Primary or less | 6451 (72.8) | 5807 (65.6) | 5379 (60.8) | 5373 (60.7) |  |
| Junior high or above | 2407 (27.2) | 3047 (34.4) | 3469 (39.2) | 3481 (39.3) |  |
| Occupation |  |  |  |  | <0.001 |
| Agriculture | 4863 (54.9) | 5195 (58.7) | 5355 (60.5) | 5372 (60.7) |  |
| Housework | 647 (7.3) | 781 (8.8) | 774 (8.7) | 1063 (12.0) |  |
| Retired | 1849 (20.9) | 1695 (19.1) | 1699 (19.2) | 1450 (16.4) |  |
| Others | 1499 (16.9) | 1183 (13.4) | 1020 (11.5) | 969 (10.9) |  |
| Occupational exposure to hazardous gases |  |  |  |  | <0.001 |
| Yes | 905 (10.2) | 1122 (12.7) | 1232 (13.9) | 1514 (17.1) |  |
| No | 7953 (89.8) | 7732 (87.3) | 7616 (86.1) | 7340 (82.9) |  |
| Income per capita (¥) |  |  |  |  | <0.001 |
| <50, 000 | 5841 (65.9) | 5669 (64.0) | 5525 (62.4) | 5429 (61.3) |  |
| ≥50, 000 | 3017 (34.1) | 3185 (36.0) | 3323 (37.6) | 3425 (38.7) |  |
| Smoking status |  |  |  |  | <0.001 |
| Yes | 4120 (46.5) | 3922 (44.3) | 3552 (40.1) | 2493 (28.2) |  |
| No | 4738 (53.5) | 4932 (55.7) | 5296 (59.9) | 6361 (71.8) |  |
| SBP (mmHg) | 132.22 (14.68) | 131.92 (14.06) | 131.36 (13.93) | 130.69 (13.03) | <0.001 |
| DBP (mmHg) | 77.78 (10.05) | 77.52 (9.53) | 77.39 (9.36) | 77.26 (9.20) | 0.002 |
| Heart rate (times/min) | 74.41 (9.94) | 74.80 (10.15) | 75.06 (9.80) | 75.29 (9.95) | <0.001 |
| SpO_2_(%) | 95.62 (5.77) | 95.91 (5.58) | 95.99 (5.62) | 96.06 (5.56) | <0.001 |
| BMI (kg/m^2^) | 23.40 (3.23) | 23.48 (3.09) | 23.51 (3.04) | 23.57 (3.09) | 0.003 |
| WC (cm) | 82.85 (9.02) | 82.93 (8.87) | 82.51 (8.54) | 82.04 (8.45) | <0.001 |
| Obesity |  |  |  |  | 0.027 |
| Normal | 5342 (60.3) | 5323 (60.1) | 5257 (59.4) | 5199 (58.7) |  |
| Overweight | 689 (7.8) | 656 (7.4) | 610 (6.9) | 658 (7.4) |  |
| Obesity | 2827 (31.9) | 2875 (32.5) | 2981 (33.7) | 2997 (33.8) |  |
| Abdominal obesity |  |  |  |  | 0.200 |
| Normal | 6476 (73.1) | 6542 (73.9) | 6587 (74.4) | 6501 (73.4) |  |
| Abdominal Obesity | 2382 (26.9) | 2312 (26.1) | 2261 (25.6) | 2353 (26.6) |  |

Abbreviation: BMI, body mass index; WC, waist circumference; SBP, systolic blood pressure; DBP, diastolic blood pressure; SpO₂, peripheral oxygen saturation; FVC%pred, forced vital capacity percent predicted; FEV₁%pred, forced expiratory volume in 1 second percent predicted; PEF%pred, peak expiratory flow percent predicted.

Values were means ± SD or n (percentages).

Table S4. Characteristics of Participants by CMM

|  | Non-CMM | CMM | *P* |
| --- | --- | --- | --- |
| N (%) | 35053 (98.98) | 361 (1.02) |  |
| Age, years | 67.40 (4.72) | 68.90 (4.14) | <0.001 |
| Sex |  |  | <0.001 |
| Male | 19586 (55.9) | 236 (65.4) |  |
| Female | 15467 (44.1) | 125 (34.6) |  |
| Education |  |  | 0.004 |
| Primary or less | 22802 (65.1) | 208 (57.6) |  |
| Junior high or above | 12251 (34.9) | 153 (42.4) |  |
| Occupation |  |  | <0.001 |
| Agriculture | 20630 (58.9) | 155 (42.9) |  |
| Housework | 3222 (9.2) | 43 (11.9) |  |
| Retired | 6580 (18.8) | 113 (31.3) |  |
| Others | 4621 (13.2) | 50 (13.9) |  |
| Occupational exposure to hazardous gases |  |  | 0.032 |
| Yes | 4710 (13.4) | 63 (17.5) |  |
| No | 30343 (86.6) | 298 (82.5) |  |
| Income per capita (¥) |  |  | 0.070 |
| <50, 000 | 22252 (63.5) | 212 (58.7) |  |
| ≥50, 000 | 12801 (36.5) | 149 (41.3) |  |
| Smoking status |  |  | 0.002 |
| Yes | 13914 (39.7) | 173 (47.9) |  |
| No | 21139 (60.3) | 188 (52.1) |  |
| SBP (mmHg) | 131.54 (13.96) | 132.42 (12.81) | 0.233 |
| DBP (mmHg) | 77.50 (9.56) | 76.36 (8.08) | 0.024 |
| Heart rate (times/min) | 74.89 (9.96) | 75.09 (10.59) | 0.706 |
| SpO_2_(%) | 95.90 (5.63) | 95.52 (6.42) | 0.200 |
| BMI (kg/m^2^) | 23.48 (3.11) | 24.38 (3.34) | <0.001 |
| WC (cm) | 82.55 (8.72) | 85.72 (9.77) | <0.001 |
| Obesity |  |  | <0.001 |
| Normal | 20948 (59.8) | 173 (47.9) |  |
| Overweight | 2567 (7.3) | 46 (12.7) |  |
| Obesity | 11538 (32.9) | 142 (39.3) |  |
| Abdominal obesity |  |  | <0.001 |
| Normal | 25886 (73.8) | 220 (60.9) |  |
| Abdominal Obesity | 9167 (26.2) | 141 (39.1) |  |
| FVC%pred | 94.06 (32.57) | 88.83 (20.96) | 0.002 |
| FEV_1_%pred | 96.66 (54.14) | 92.59 (21.93) | 0.153 |
| PEF%pred | 80.79 (65.27) | 75.69 (22.38) | 0.138 |
| Lung function category |  |  | <0.001 |
| Preserved Lung function | 27242 (77.7) | 242 (67.0) |  |
| Restrictive spirometry | 7811 (22.3) | 119 (33.0) |  |

Abbreviation: BMI, body mass index; WC, waist circumference; SBP, systolic blood pressure; DBP, diastolic blood pressure; SpO₂, peripheral oxygen saturation; FVC%pred, forced vital capacity percent predicted; FEV₁%pred, forced expiratory volume in 1 second percent predicted; PEF%pred, peak expiratory flow percent predicted.

Values were means ± SD or n (percentages).

Table S5. Comparison of Characteristics Between Included and Excluded Participants

|  | Included (n=35414) | Excluded (n=25472) | *P* |
| --- | --- | --- | --- |
| Age, years | 67.41 (4.72) | 68.97 (4.20) | <0.001 |
| Sex |  |  |  |
| Male | 19822 (56.0) | 16941 (66.6) | <0.001 |
| Female | 15592 (44.0) | 8489 (33.4) |  |
| Education |  |  |  |
| Primary or less | 23010 (65.0) | 17968 (70.5) | <0.001 |
| Junior high or above | 12404 (35.0) | 7504 (29.5) |  |
| Occupation |  |  |  |
| Agriculture | 20785 (58.7) | 17279 (67.8) | <0.001 |
| Housework | 3265 (9.2) | 2582 (10.1) |  |
| Retired | 6693 (18.9) | 3132 (12.3) |  |
| Others | 4671 (13.2) | 2479 (9.7) |  |
| Income per capita (¥) |  |  |  |
| <50, 000 | 22464 (63.4) | 17635 (69.2) | <0.001 |
| ≥50, 000 | 12950 (36.6) | 7837 (30.8) |  |
| Smoking status |  |  |  |
| Yes | 14087 (39.8) | 13244 (52.1) | <0.001 |
| No | 21327 (60.2) | 12186 (47.9) |  |
| Occupational exposure to hazardous gases |  |  |  |
| Yes | 4773 (13.5) | 3332 (13.1) | 0.159 |
| No | 30641 (86.5) | 22140 (86.9) |  |
| BMI (kg/m^2^) | 23.49 (3.12) | 22.83 (3.16) | <0.001 |
| WC (cm) | 82.58 (8.73) | 82.01 (9.13) | <0.001 |
| SBP (mmHg) | 131.55 (13.95) | 132.62 (14.83) | <0.001 |
| DBP (mmHg) | 77.49 (9.54) | 78.25 (9.94) | <0.001 |
| Heart rate (times/min) | 74.89 (9.97) | 74.66 (11.01) | 0.008 |
| SpO_2_(%) | 95.89 (5.63) | 95.87 (6.18) | 0.637 |
| FVC%pred | 94.00 (32.48) | 95.99 (24.94) | 0.089 |
| FEV_1_%pred | 96.62 (53.91) | 97.94 (26.02) | 0.495 |
| PEF%pred | 80.74 (64.98) | 82.69 (25.51) | 0.401 |

Abbreviation: BMI, body mass index; WC, waist circumference; SBP, systolic blood pressure; DBP, diastolic blood pressure; SpO₂, peripheral oxygen saturation; FVC%pred, forced vital capacity percent predicted; FEV₁%pred, forced expiratory volume in 1 second percent predicted; PEF%pred, peak expiratory flow percent predicted.

Values were means ± SD or n (percentages).

Table S6. Associations of Lung Function with Cardiometabolic Multimorbidity Using Tertile Categories

| Lung function | Model 1 | Model 2 | Model 3 | Model 4 |
| --- | --- | --- | --- | --- |
|  | OR (95%CI) | OR (95%CI) | OR (95%CI) | OR (95%CI) |
| FVC%pred |  |  |  |  |
| Tertile1 | Reference | Reference | Reference | Reference |
| Tertile2 | **0.68 (0.53, 0.86)** | **0.71 (0.55, 0.90)** | **0.70 (0.54, 0.89)** | **0.72 (0.56, 0.92)** |
| Tertile3 | **0.55 (0.42, 0.71)** | **0.58 (0.45, 0.75)** | **0.58 (0.44, 0.75)** | **0.62 (0.47, 0.80)** |
| *P* for trend | **<0.001** | **<0.001** | **<0.001** | **<0.001** |
| FEV_1_%pred |  |  |  |  |
| Tertile1 | Reference | Reference | Reference | Reference |
| Tertile2 | 0.80 (0.63, 1.02) | 0.83 (0.65, 1.05) | 0.81 (0.64, 1.04) | 0.85 (0.67, 1.09) |
| Tertile3 | **0.61 (0.47, 0.80)** | **0.62 (0.48, 0.81)** | **0.62 (0.48, 0.81)** | **0.66 (0.51, 0.86)** |
| *P* for trend | **<0.001** | **<0.001** | **<0.001** | **0.002** |
| PEF%pred |  |  |  |  |
| Tertile1 | Reference | Reference | Reference | Reference |
| Tertile2 | **0.76 (0.59, 0.97)** | 0.80 (0.62, 1.02) | 0.79 (0.61, 1.01) | 0.79 (0.61, 1.01) |
| Tertile3 | **0.68 (0.53, 0.88)** | **0.76 (0.59, 0.98)** | **0.74 (0.57, 0.95)** | **0.74 (0.57, 0.96)** |
| *P* for trend | **0.002** | **0.031** | **0.017** | **0.020** |

Model 1: unadjusted; Model 2: adjust for age and sex; Model 3: adjust for Model 2 plus education, occupation, income per capita, smoking status, occupational exposure to hazardous gases; Model 4: adjust for Model 3 plus obesity, abdominal obesity, SBP, DBP, Heart rate, and SpO_2_.

Table S7. Associations of Lung Function with Cardiometabolic Multimorbidity Using Quintile Categories

| Lung function | Model 1 | Model 2 | Model 3 | Model 4 |
| --- | --- | --- | --- | --- |
|  | OR (95%CI) | OR (95%CI) | OR (95%CI) | OR (95%CI) |
| FVC%pred |  |  |  |  |
| Quintile1 | Reference | Reference | Reference | Reference |
| Quintile2 | 0.81 (0.60, 1.07) | 0.84 (0.62, 1.12) | 0.82 (0.61, 1.10) | 0.83 (0.62, 1.11) |
| Quintile3 | **0.66 (0.48, 0.89)** | **0.70 (0.51, 0.95)** | **0.68 (0.50, 0.93)** | **0.71 (0.52, 0.96)** |
| Quintile4 | **0.47 (0.33, 0.65)** | **0.50 (0.35, 0.70)** | **0.49 (0.34, 0.69)** | **0.52 (0.37, 0.73)** |
| Quintile5 | **0.53 (0.38, 0.73)** | **0.57 (0.40, 0.78)** | **0.56 (0.40, 0.78)** | **0.60 (0.43, 0.84)** |
| *P* for trend | **<0.001** | **<0.001** | **<0.001** | **<0.001** |
| FEV_1_%pred |  |  |  |  |
| Quintile1 | Reference | Reference | Reference | Reference |
| Quintile2 | 0.88 (0.65, 1.17) | 0.90 (0.67, 1.21) | 0.90 (0.67, 1.20) | 0.93 (0.69, 1.25) |
| Quintile3 | **0.66 (0.48, 0.90)** | **0.69 (0.50, 0.94)** | **0.67 (0.49, 0.92)** | **0.71 (0.52, 0.98)** |
| Quintile4 | **0.58 (0.41, 0.80)** | **0.60 (0.43, 0.83)** | **0.59 (0.42, 0.82)** | **0.64 (0.46, 0.89)** |
| Quintile5 | **0.56 (0.40, 0.77)** | **0.57 (0.40, 0.79)** | **0.57 (0.40, 0.79)** | **0.61 (0.43, 0.85)** |
| *P* for trend | **<0.001** | **<0.001** | **<0.001** | **<0.001** |
| PEF%pred |  |  |  |  |
| Quintile1 | Reference | Reference | Reference | Reference |
| Quintile2 | **0.71 (0.52, 0.97)** | 0.74 (0.54, 1.01) | 0.74 (0.54, 1.00) | 0.74 (0.54, 1.00) |
| Quintile3 | **0.69 (0.50, 0.94)** | 0.75 (0.54, 1.02) | 0.73 (0.53, 1.00) | 0.74 (0.54, 1.01) |
| Quintile4 | 0.75 (0.55, 1.02) | 0.83 (0.61, 1.12) | 0.81 (0.60, 1.11) | 0.82 (0.60, 1.12) |
| Quintile5 | **0.52 (0.37, 0.72)** | **0.60 (0.42, 0.84)** | **0.57 (0.40, 0.81)** | **0.58 (0.40-0.81)** |
| *P* for trend | **<0.001** | **0.010** | **0.005** | **0.007** |

Model 1: unadjusted; Model 2: adjust for age and sex; Model 3: adjust for Model 2 plus education, occupation, income per capita, smoking status, occupational exposure to hazardous gases; Model 4: adjust for Model 3 plus obesity, abdominal obesity, SBP, DBP, Heart rate, and SpO_2_.

Table S8. Association Between Lung Function Z-scores (GLI Reference Equations) and Cardiometabolic Multimorbidity

| Lung function | Model 1 | Model 2 | Model 3 | Model 4 |
| --- | --- | --- | --- | --- |
|  | OR (95%CI) | OR (95%CI) | OR (95%CI) | OR (95%CI) |
| FVC Z-score | 0.93 (0.89, 0.96) | 0.85 (0.76, 0.96) | 0.84 (0.75, 0.95) | 0.86 (0.76, 0.97) |
| FEV₁ Z-score | 0.92 (0.89, 0.95) | 0.83 (0.75, 0.93) | 0.82 (0.74, 0.92) | 0.83 (0.75, 0.93) |

Z-scores were calculated using the GLI-2012 reference equations for Asian populations.

Model 1: unadjusted; Model 2: adjust for age and sex; Model 3: adjust for Model 2 plus education, occupation, income per capita, smoking status, occupational exposure to hazardous gases; Model 4: adjust for Model 3 plus obesity, abdominal obesity, SBP, DBP, Heart rate, and SpO_2_.

Table S9. Associations of Lung Function and Lung Function Category with Cardiometabolic Multimorbidity (Including Hypertension in CMM Definition)

| Lung function | Case/Total | Model 1 | Model 2 | Model 3 | Model 4 |
| --- | --- | --- | --- | --- | --- |
|  |  | OR (95%CI) | OR (95%CI) | OR (95%CI) | OR (95%CI) |
| FVC%pred |  |  |  |  |  |
| Quartile1 | 128/8865 | Reference | Reference | Reference | Reference |
| Quartile2 | 94/8842 | 0.86 (0.73-1.01) | 0.90 (0.77-1.05) | 0.88 (0.75-1.03) | 0.89 (0.76-1.05) |
| Quartile3 | 71/8857 | **0.75 (0.64-0.89)** | **0.79 (0.67-0.94)** | **0.78 (0.66-0.92)** | **0.82 (0.69-0.96)** |
| Quartile4 | 68/8850 | **0.68 (0.58-0.81)** | **0.73 (0.61-0.86)** | **0.71 (0.60-0.85)** | **0.77 (0.65-0.91)** |
| *P* for trend |  | **<0.001** | **<0.001** | **<0.001** | **0.001** |
| FEV_1_%pred |  |  |  |  |  |
| Quartile1 | 114/8855 | Reference | Reference | Reference | Reference |
| Quartile2 | 70/8884 | 0.86 (0.73-1.01) | 0.89 (0.75-1.04) | 0.87 (0.74-1.03) | 0.90 (0.77-1.06) |
| Quartile3 | 75/8824 | **0.79 (0.67-0.93)** | **0.82 (0.70-0.97)** | **0.81 (0.69-0.96)** | 0.86 (0.73-1.02) |
| Quartile4 | 119/8851 | **0.74 (0.63-0.88)** | **0.75 (0.63-0.88)** | **0.74 (0.63-0.88)** | **0.79 (0.67-0.94)** |
| *P* for trend |  | **<0.001** | **<0.001** | **<0.001** | **0.007** |
| PEF%pred |  |  |  |  |  |
| Quartile1 | 119/8858 | Reference | Reference | Reference | Reference |
| Quartile2 | 86/8854 | **0.83 (0.70-0.98)** | 0.87 (0.73-1.02) | 0.86 (0.73-1.01) | 0.86 (0.73-1.02) |
| Quartile3 | 87/8848 | **0.84 (0.71-0.99)** | 0.90 (0.77-1.07) | 0.89 (0.75-1.05) | 0.90 (0.76-1.06) |
| Quartile4 | 69/8854 | **0.80 (0.68-0.95)** | 0.91 (0.77-1.08) | 0.88 (0.74-1.04) | 0.89 (0.75-1.05) |
| *P* for trend |  | **0.012** | 0.301 | 0.150 | 0.194 |
| Lung function category |  |  |  |  |  |
| Restrictive spirometry | 242/27484 | Reference | Reference | Reference | Reference |
| Preserved Lung function | 119/7930 | **0.74 (0.65-0.85)** | **0.78 (0.69-0.90)** | **0.77 (0.67-0.88)** | **0.80 (0.70-0.92)** |

Model 1: adjust for none; Model 2: adjust for age and sex; Model 3: adjust for Model 2 plus education, occupation, income per capita, smoking status, occupational exposure to hazardous gases; Model 4: adjust for Model 3 plus obesity, abdominal obesity, SBP, DBP, Heart rate, and SpO_2_.

Table 10. Associations of General Obesity and Abdominal obesity with Cardiometabolic Multimorbidity (Including Hypertension in CMM Definition)

| Obesity | Case/Total | Model 1 | Model 2 | Model 3 | Model 3 |
| --- | --- | --- | --- | --- | --- |
|  |  | OR (95%CI) | OR (95%CI) | OR (95%CI) | OR (95%CI) |
| General Obesity |  |  |  |  |  |
| Obesity | 142/11680 | Reference | Reference | Reference | Reference |
| Overweight | 46/2613 | **0.48 (0.39-0.58)** | **0.45 (0.37-0.55)** | **0.46 (0.38-0.56)** | **0.47 (0.39-0.57)** |
| Normal | 173/21121 | **0.74 (0.61-0.90)** | **0.72 (0.59-0.88)** | **0.71 (0.59-0.87)** | **0.72 (0.59-0.88)** |
| BMI (Per-SD) |  | **1.32 (1.25-1.39)** | **1.34 (1.27-1.42)** | **1.34 (1.26-1.41)** | **1.33 (1.25-1.40)** |
| Abdominal obesity |  |  |  |  |  |
| No | 220/26106 | Reference | Reference | Reference | Reference |
| Yes | 141/9308 | **0.53 (0.47-0.60)** | **0.52 (0.46-0.59)** | **0.53 (0.47-0.60)** | **0.53 (0.47-0.60)** |
| WC (Per-SD) |  | **1.40 (1.33-1.48)** | **1.38 (1.31-1.46)** | **1.37 (1.30-1.45)** | **1.36 (1.29-1.44)** |

Model 1: adjust for none; Model 2: adjust for age and sex; Model 3: adjust for Model 2 plus education, occupation, income per capita, smoking status, occupational exposure to hazardous gases; Model 4: adjust for Model 3 plus Lung function category, SBP, DBP, Heart rate, and SpO_2_.

Table 11. Joint effect of Lung function category and Obesity on Cardiometabolic Multimorbidity (Including Hypertension in CMM Definition)

| Lung function category | Obesity | Case/Total | OR (95%CI) | *P* for interaction |
| --- | --- | --- | --- | --- |
| Lung function category + General obesity | | | | 0.314 |
| Restrictive spirometry | Overweight and obesity | 165/3421 | Reference |  |
| Restrictive spirometry | Normal | 146/4509 | **0.67 (0.53-0.84)** |  |
| Preserved Lung function | Overweight and obesity | 421/10872 | 0.84 (0.70-1.01) |  |
| Preserved Lung function | Normal | 387/16612 | **0.49 (0.41-0.60)** |  |
| Lung function category + Abdominal obesity | | | | 0.551 |
| Restrictive spirometry | Abdominal obesity | 131/2295 | Reference |  |
| Restrictive spirometry | Normal | 180/5635 | **0.57 (0.45-0.72)** |  |
| Preserved Lung function | Abdominal obesity | 314/7013 | 0.84 (0.68-1.04) |  |
| Preserved Lung function | Normal | 494/20471 | **0.44 (0.36-0.54)** |  |

Adjust for sex, age, education, occupation, income per capita, smoking status, occupational exposure to hazardous gases, obesity, abdominal obesity, SBP, DBP, Heart rate, and SpO_2_.

Figure S1.


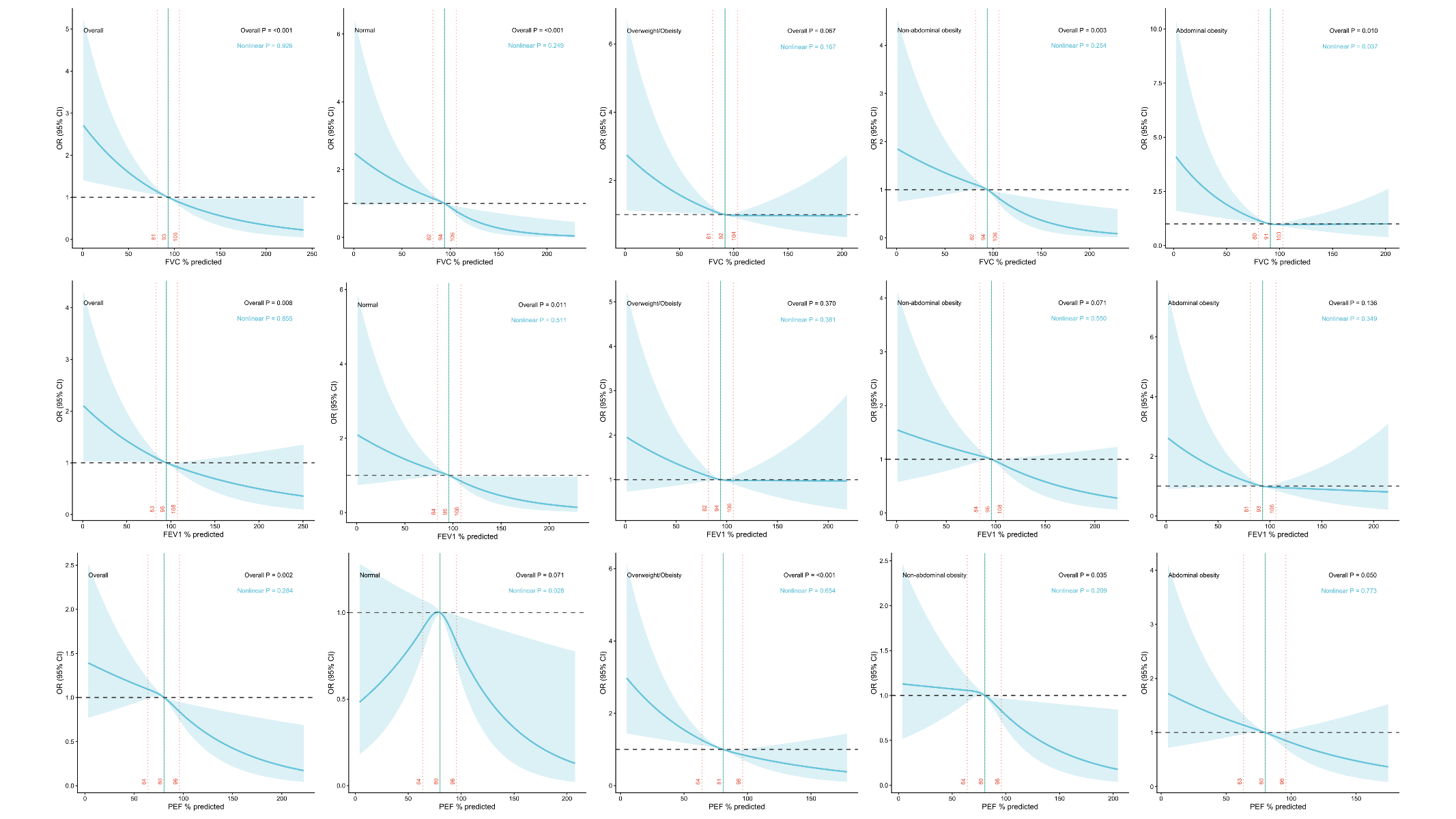

Supplement: Supplementary file 1 [file Data_Sheet_1.docx]
